# Prehospital traumatic cardiac arrest: a systematic review and meta-analysis

**DOI:** 10.1007/s00068-022-01941-y

**Published:** 2022-03-25

**Authors:** Niek Johannes Vianen, Esther Maria Maartje Van Lieshout, Iscander Michael Maissan, Wichor Matthijs Bramer, Dennis Den Hartog, Michael Herman Jacob Verhofstad, Mark Gerrit Van Vledder

**Affiliations:** 1 Trauma Research Unit, Department of Surgery, Erasmus MC, University Medical Center Rotterdam, P.O. Box 2040, 3000 CA Rotterdam, The Netherlands; 2 Department of Anesthesiology, Erasmus University Medical Center Rotterdam, Rotterdam, The Netherlands; 3 Medical Library, Erasmus MC, Erasmus University Medical Centre Rotterdam, 3000 CS Rotterdam, The Netherlands

**Keywords:** Traumatic cardiac arrest (TCA), Mortality, Neurological outcome, Registry type, Organization of EMS system, Prognostic factors

## Abstract

**Background:**

Circulatory arrest after trauma is a life-threatening situation that mandates urgent action. The aims of this systematic review and meta-analysis on prehospital traumatic cardiac arrest (TCA) were to provide an updated pooled mortality rate for prehospital TCA, to investigate the impact of the time of patient inclusion and the type of prehospital trauma system on TCA mortality rates and neurological outcome, and to investigate which pre- and intra-arrest factors are prognostic for prehospital TCA mortality.

**Methods:**

This review was conducted in accordance with the PRISMA and CHARMS guidelines. Databases were searched for primary studies published about prehospital TCA patients (1995–2020). Studies were divided into various EMS-system categories. Data were analyzed using MedCalc, Review Manager, Microsoft Excel, and Shinyapps Meta Power Calculator software.

**Results:**

Thirty-six studies involving 51.722 patients were included. Overall mortality for TCA was 96.2% and a favorable neurological outcome was seen in 43.5% of the survivors. Mortality rates were 97.2% in studies including prehospital deaths and 92.3% in studies excluding prehospital deaths. Favorable neurological outcome rates were 35.8% in studies including prehospital deaths and 49.5% in studies excluding prehospital deaths. Mortality rates were 97.6% if no physician was available at the prehospital scene and 93.9% if a physician was available. Favorable neurological outcome rates were 57.0% if a physician was available on scene and 38.0% if no physician was available. Only non-shockable rhythm was associated with a higher mortality (RR 1.12, *p* = 0.06).

**Conclusion:**

Approximately 1 in 20 patients with prehospital TCA will survive; about 40% of survivors have favorable neurological outcome.

**Supplementary Information:**

The online version contains supplementary material available at 10.1007/s00068-022-01941-y.

## Introduction

Circulatory arrest after trauma is a severe and life-threatening situation that mandates urgent action. Over the past years, the prehospital management of patients with traumatic cardiac arrest (TCA) has received much attention in international scientific literature. Multiple authors have reported on survival rates and prognostic factors for patients with prehospital traumatic cardiac arrest, with survival rates ranging from 0% to almost 27% in individual reports [1–3]. A 2012 systematic review including 47 studies published between 1982 and 2010 reported a pooled 3.3% survival rate among adults [4]. In a more recent systematic review and meta-analysis on prognostic factors associated with survival following TCA, published in 2020, including 53 studies published between 1982 and 2019, cardiac motion on ultrasound and a shockable rhythm on first ECG were associated with increased odds of survival in a pooled unadjusted analysis [5].

While both reviews provide insightful information regarding survival and prognostic factors for survival in these severely injured patients, there is more to be told regarding these aggregated data. First, neither of the mentioned reviews considers that several of the included studies have included patients that died at the scene of the accident, where others do not, potentially confounding any assumptions made regarding survival rates as well as prognostic factors. Second, neither of these reviews has investigated whether the level of training of prehospital emergency care providers does impact on survival rates in prehospital TCA: where some prehospital trauma systems have specialized Advanced Cardiovascular Life Support (ACLS) and/or prehospital Advanced Trauma Life Support (PHTLS) registered nurses and/or physicians readily available in each case of prehospital TCA, other systems rely on emergency medicine technicians providing only basic life support on-scene. As opportunities for prehospital resuscitative procedures rely on the level of training of prehospital caregivers, we hypothesize that this may also impact on survival rates [6]. Since the rate of survival seems to be the driving force in any dialogue involving prehospital management of patients in TCA, there should be absolute clarity regarding the type of data used to obtain aggregated survival rates and prognostic factors and how these are impacted by the moment of patient inclusion and type of prehospital trauma system.

Therefore, the aims of this current review and meta-analysis on prehospital TCA were (1) to provide an updated pooled mortality rate for prehospital TCA, (2) to investigate the impact of the moment of patient inclusion and the type of prehospital emergency trauma system on TCA mortality rates and neurological outcome, and (3) to investigate which pre- and intra-arrest factors are prognostic factors for prehospital TCA mortality.

## Methods

### Data sources

The methods in this review are described based on the *preferred reporting items for systematic reviews and meta-analyses* (Prisma) Checklist [7] and the Prisma-S extension to the PRISMA Statement for Reporting Literature Searches in Systematic Reviews [8]. The search was developed in Embase.com, optimized for sensitivity and then translated to other databases following the method as described before [9]. The search was carried out in the databases Embase.com, Medline ALL via Ovid, Web of Science Core Collection (Science Citation Index Expanded; Social Sciences Citation Index; Arts & Humanities Citation Index; Conference Proceedings Citation Index-Science; Conference Proceedings Citation Index-Social Science & Humanities and Emerging Sources Citation Index and the Cochrane Central Register of Controlled Trials via Wiley). Additionally, a search was performed in Google Scholar where the 200 top relevant references were downloaded. After the original search was performed in March 2019, the search was updated twice (last update; July 13, 2020). The references were imported into EndNote and duplicates were removed as described previously [10, 11].

### Search strategy

The search strategies for Embase and Medline used relevant thesaurus terms from Emtree and Medical Subject Headings (MeSH) respectively. In all databases terms were searched in titles and abstracts of references. The search contained terms for (1) prehospital cardiac arrest or prehospital Advanced Life Support, and (2) injuries and trauma. Terms were combined with Boolean operators AND and OR and proximity operators were used to combined terms into phrases. The full search strategies of all databases are available in the appendix. The searches in Embase and Web of Science were limited to exclude conference papers. The reference lists of retrieved non-included relevant review articles and of the included references have been scanned for relevant references missed by the search. No authors or subject experts were contacted, and unindexed journals in the field were not browsed.

### Study selection criteria

Studies meeting the following criteria were included: (1) articles written in English, French, German, or Dutch, and (2) studies with reported outcomes of interest for prehospital TCA. Exclusion criteria were: (1) (> 10%) pediatric patients, (2) military report or combat patients, (3) studies evaluating only a specific treatment (e.g., thoracotomy or REBOA), (4) letters to the editor, expert opinions, systematic reviews, and meta-analyses, (5) animal studies, (6) articles for which the full text was not available to the researchers, and (7) studies published before 1995. Two authors independently (NJV and MGVV) screened the titles and abstracts for relevance and then extracted and selected relevant full text records, where possible. Subsequently, any leftover duplicates were removed, and the full texts of the selected articles were assessed. Discrepancies were resolved through discussion at each stage.

Studies were assessed on quality using the Newcastle–Ottawa Quality Assessment Scale (NOS) [12]. No stars were awarded for comparability since the current literature is divided whether there are prognostic factors that need to be adjusted for. Follow up was rated sufficient if patients were followed up for at least 14 days or were followed up until discharge from hospital. The loss to follow up cut-off point was set on 2.5%. In the Newcastle–Ottawa Quality Assessment Scale, studies are awarded stars based on their quality. The maximum of achievable stars was seven, and studies with less than six stars were excluded.

The risk of bias was assessed using the RevMan Risk of Bias Tool, were all included studies were scored for different types of bias. A study was excluded if it scored either a high risk of bias in one or more categories or if it scored an unknown risk of bias in two or more categories.

### Data extraction

Data were extracted by two researchers independently (NJV and MGVV) using a data collection sheet. Any discrepancy was resolved by discussion. The following variables were extracted: author name, year of publication, journal name, country of patient inclusion, start and end date of patient inclusion, study design, study database, database registry type (Studies including prehospital deaths and studies excluding prehospital deaths; Studies from prehospital registries and studies from hospital registries where prehospital care providers are not allowed to declare a patient dead on scene and thus every patient is transported to a hospital were marked as “study including prehospital deaths”), organization of prehospital EMS system (physician or no physician), number of patients included, patient characteristics (sex, age), trauma type (penetrating, blunt, road traffic accident, fall from height), arrest characteristics (witnessed, unwitnessed, bystander CPR), first monitored rhythm (shockable, non-shockable), prehospital interventions (intubation, administration of epinephrine), survival rates, and long-term neurological outcome. Favorable neurological outcome was defined as a Cerebral Performance Category (CPC) I or II, or as a Glasgow Outcome Scale (GOS) 4 or 5 [13, 14]. The database registry types were divided into two predefined categories: (1) studies using databases including patients declared dead on-scene, (2) studies using databases excluding patients declared dead on-scene. Studies from EMS systems where patients can only be declared dead by an ER-physician and patients are thus always transported to a hospital were included in the first category. Similarly, the EMS systems were divided in two predefined categories: (1) studies from countries or regions with a physician-based EMS service and (2) studies from countries or regions without the availability of a HEMS / EMS physician.

### Data analysis

Data regarding registry type and TCA survival, and registry type and neurological outcome were pooled using MedCalc Statistical Software version 18.2.1 (MedCalc Software bvba, Ostend, Belgium; http://www.medcalc.org). Likewise, the data regarding organization of EMS system and TCA survival, and organization of EMS system and neurological outcome are analyzed using MedCalc. Data regarding prognostic factors for TCA survival were analyzed using Review Manager statistical software (RevMan 5.4.1, The Nordic Cochrane Collaboration, Copenhagen, Denmark).

Heterogeneity was assessed using a *Q*-test and *I*^2^ statistic. In MedCalc, the *Q*-test was used as a binary test to investigate the presence of statistically significant heterogeneity, and the *I*^2^ statistic as a quantitative measure of heterogeneity. In RevMan, the Chi^2^ test was used as a binary test to investigate the presence of statistically significant heterogeneity, and again the *I*^2^ statistic as a quantitative measure of heterogeneity. In the *I*^2^ statistic, the limit for the quantitative measure of heterogeneity was set at 40%, with values above 40% set as any significant level of heterogeneity. Mantel–Haenszel models were applied in accordance with the heterogeneity of the data; if the result of the *I*^2^ statistic was below 40%, fixed effect models were used and if the result of the *I*^2^ statistic was above 40%, random effect models were used.

Data are reported as pooled estimate or risk ratio with corresponding 95% confidence interval (95% CI), as applicable. Forrest plots and funnel plots are shown.

RevMan funnel plots are evaluated using Egger’s regression test to investigate if there is a significant amount of publication bias within the analysis, Meta-Essentials version 1.50 in Microsoft Excel was used to perform this analysis [15] (Microsoft Excel 2016, Microsoft Corporation, Redmond, United States of America, http://office.microsoft.com/excel).

A power analysis on the prognostic factors analysis was performed using the Shinyapps Meta Power Calculator (Shinyapps, RStudio, Boston, United States of America, http://jtiebel.shinyapps.io/MetaPowerCalculator).

### Medical ethical approval

As systematic reviews and meta-analysis are exempt of IRB approval in the Netherlands, no medical ethical approval was needed to conduct this study.

## Results

### Search results

**Fig. 1:**
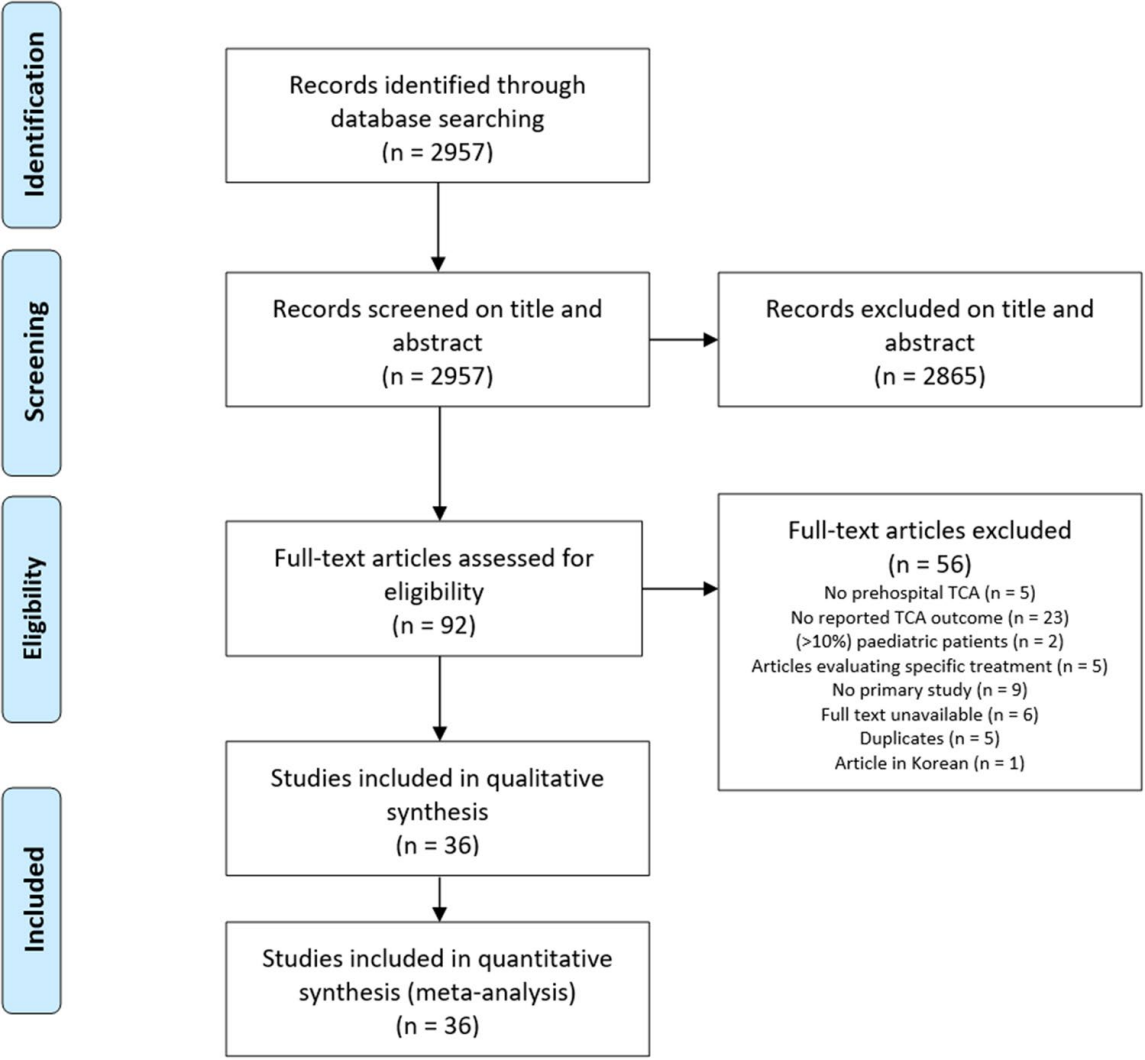
PRISMA flow diagram

We found 2957 articles, of which 2865 were excluded based on title and/or abstract. The remaining 92 articles were then screened on full text, if available. Fifty-six articles were excluded for various reasons (Fig. 1), resulting in 36 articles being included [1–3, 5, 6, 16–47]. All studies scored 6 or 7 points on the Newcastle–Ottawa Quality Assessment Scale and therefore no study was excluded for quality reasons (Table 1). Likewise, no studies were excluded after the risk of bias evaluation, since no study scored either a high risk of bias in one or more categories, or an unknown bias risk in two or more categories. The risk of bias evaluation is displayed as a RevMan Risk of Bias Summary Tool and funnel plots (supplemental Figs. 8–14).

**Table 1:** Results of the Newcastle–Ottawa quality assessment

	Study	Study design	Selection				Comparability	Exposure/Outcome			Result
			1	2	3	4	1/2	1	2	3	
1	Aoki et al. [39]	Retrospective cohort study with post-hoc case–control analysis	1	1	1	1	0	1	1	1	7
2	Barnard et al. [28]	Retrospective cohort	1	1	1	1	0	1	1	1	7
3	Barnard et al. [40]	Retrospective cohort	1	1	1	1	0	1	1	1	7
4	Beck et al. [29]	Retrospective cohort	1	1	1	1	0	1	1	1	7
5	Beck et al. [1]	Retrospective cohort	1	1	1	1	0	1	1	1	7
6	Chen et al. [41]	Retrospective cohort	1	1	1	1	0	1	1	1	7
7	Chia et al. [30]	Retrospective cohort	1	1	1	1	0	1	1	1	7
8	Chiang et al. [31]	Retrospective cohort	1	1	1	1	0	1	1	1	7
9	Claesson et al. [32]	Retrospective cohort	1	1	1	1	0	1	1	1	7
10	Cureton et al. [23]	Retrospective cohort	1	1	1	1	0	1	1	1	7
11	David et al. [19]	Randomized controlled trial	1	0	1	1	0	1	1	1	6
12	Deasy et al. [24]	Retrospective cohort	1	1	1	1	0	1	1	1	7
13	Di Bartolomeo et al. [6]	Prospective population-based study	1	1	1	1	0	1	1	1	7
14	Djarv et al. [35]	Retrospective cohort	1	1	1	1	0	1	1	1	7
15	Duchateau et al. [33]	Retrospective cohort	1	1	1	1	0	1	1	1	7
16	Escutnaire et al. [36]	Retrospective cohort	1	1	1	1	0	1	1	1	7
17	Evans et al. [27]	Retrospective cohort	1	1	1	1	0	1	1	1	7
18	Faucher et al. [21]	Retrospective cohort	1	1	1	1	0	1	1	1	7
19	Fukuda et al. [37]	Retrospective cohort	1	1	1	1	0	1	1	1	7
20	Gräsner et al. [2]	Retrospective cohort	1	1	1	1	0	1	1	1	7
21	Huber-Wagner et al. [20]	Retrospective cohort	1	1	1	1	0	1	1	1	7
22	Irfan et al. [34]	Retrospective cohort	1	1	1	1	0	1	1	1	7
23	Israr et al. [42]	Retrospective cohort	1	1	1	1	0	1	1	1	7
24	Javaudin et al. [46]	Retrospective cohort	1	1	1	1	0	1	1	1	7
25	Jun et al. [47]	Retrospective cohort	1	1	1	1	0	1	1	1	7
26	Kitamura et al. [26]	Retrospective cohort	1	1	1	1	0	1	1	1	7
27	Lin et al. [25]	Retrospective cohort	1	1	1	1	0	1	1	1	7
28	Lockey et al. [3]	Retrospective cohort	1	1	1	1	0	1	1	0	6
29	Lu et al. [43]	Retrospective cohort	1	1	1	1	0	1	1	1	7
30	Moriwaki et al. [22]	Retrospective cohort	1	1	1	1	0	1	1	1	7
31	Pickens et al. [17]	Retrospective cohort	1	1	1	1	0	1	1	1	7
32	Stockinger et al. [16]	Retrospective cohort	1	1	1	1	0	1	1	1	7
33	ter Avest et al. [44]	Retrospective cohort	1	1	1	1	0	1	1	1	7
34	Tsutsumi et al. [38]	Retrospective cohort	1	1	1	1	0	1	1	1	7
35	Willis et al. [18]	Retrospective cohort	1	1	1	1	0	1	1	1	7
36	Yamamoto et al. [45]	Prospective observational study with post hoc analysis	1	1	1	1	0	1	0	1	6

1 one star awarded for criterion, 0 no star awarded for criterion. The right-most column shows the total number of stars awarded per included study

In total, 51.722 patients were involved in this systematic review and meta-analysis.

### Study characteristics

Most included studies (*n* = 21) were published between 2016 and 2019 [27–47], and most of the 36 primary studies were carried out in either Europe (*n* = 14) [2, 3, 6, 19–21, 28, 32, 33, 35, 36, 40, 44, 46] or Asia (*n* = 13) [22, 25, 26, 30, 31, 34, 37–39, 41, 43, 45, 47]. Almost all included studies were retrospective cohort studies (*n* = 32) [1–3, 16–18, 20–38, 40–44, 46, 47] and included both penetrating and blunt trauma cases (*n* = 30) [1–3, 16, 17, 19–21, 23–25, 27, 28, 30–36, 40–47]. More detailed characteristics of the included primary studies are shown in Table 2.

**Table 2:** Primary study characteristics

	Study	Country	Journal	Study design	Database	Start patient inclusion	Stop patient inclusion	Total number of patients in study	Number of patients included in meta-analysis	Trauma type
1	Aoki et al. [39]	Japan	*Scientific Reports (Nature)*	Retrospective cohort study with post-hoc case–control analysis	All-Japan Utstein Registry	01–2012	12–2015	465.932	5.204	Blunt
2	Barnard et al. [28]	UK	*Resuscitation*	Retrospective cohort	TARN (Trauma Audit and Research Network	01–2009	09–2015	227.944	576	Both
3	Barnard et al. [40]	UK	*Emergency Medical Journal*	Retrospective cohort	EEAST (East of UK Ambulance Service NHS Trust)	01–2015	07–2017	9.109	304	Both
4	Beck et al. [1]	Australia	*Resuscitation*	Retrospective cohort	SJA-WHA (St John Ambulance Western Australia)	01–1997	12–2014	21.071	1.354	Both
5	Beck et al. [29]	Australia	*Emergency Medical Journal + Resuscitation*	Retrospective cohort	VACAR (Victorian Ambulance Cardiac Arrest Registry)	07–2008	06–2014	2.334	660	Both
6	Chen et al. [41]	Taiwan	*Injury*	Retrospective cohort	5 local hospitals	01–2010	12–2014	560	463	Both
7	Chia et al. [30]	Singapore, Malaysia, Japan and Thailand	*Resuscitation*	Retrospective cohort	PAROS (Pan-Asian Resuscitation Outcomes Study)	01–2009	12–2012	66.780	1.554	Both
8	Chiang et al. [31]	Taiwan	*Emergency Medical Journal*	Retrospective cohort	Taipei Fire City Department	01–2009	12–2013	921	893	Blunt
9	Claesson et al. [32]	Sweden	*Resuscitation*	Retrospective cohort	SRCR (Swedish Registry of Cardiopulmonary Resuscitation)	01–2004	12–2014	70.846	1.553	Both
10	Cureton et al. [23]	USA	*Journal of Trauma and Acute Care Surgery*	Retrospective cohort	Local hospital	01–2002	09–2008	318	318	Both
11	David et al. [19]	France and Belgium	*Critical Care Medicine*	Randomized controlled trial	12 local hospitals	01–1994	09–1996	2.910	268	Both
12	Deasy et al. [24]	Australia	*Resuscitation*	Retrospective cohort	VACAR (Victorian Ambulance Cardiac Arrest Registry)	01–2000	12–2009	33.178	2.187	Both
13	Di Bartolomeo et al. [6]	Italy	*Prehospital Emergency Care*	Prospective population-based study	Local FVG (Friuli Venezia Giulia)	01–1998	02–1999	181	129	Both
14	Djarv et al. [35]	Sweden	*Scandinavian Journal of Trauma, Resuscitation and Emergency Medicine*	Retrospective cohort	SRCR (Swedish Registry of Cardiopulmonary Resuscitation)	01–1990	12–2016	72.547	1.774	Both
15	Duchateau et al. [33]	France	*Emergency Medical Journal*	Retrospective cohort	3 local hospitals	01–2010	01–2013	88	88	Both
16	Escutnaire et al. [36]	France	*Resuscitation*	Retrospective cohort	RéAC (French National Cardiac Arrest Registry)	07–2011	01–2017	60.157	3.209	Both
17	Evans et al. [27]	USA and Canada	*Journal of Trauma Acute Care Surgery*	Retrospective cohort	ROC trauma registries	12–2005	06–2011	19.549	2.300	Both
18	Faucher et al. [21]	France	*Annales françaises d'anesthesie et de réanimation*	Retrospective cohort	Local regional database	01–2004	12–2005	1.552	129	Both
19	Fukuda et al. [37]	Japan	*JAMA Surgery*	Retrospective cohort	All-Japan Utstein Registry	01–2013	12–2014	251.075	4.382	Blunt
20	Gräsner et al. [2]	Germany	*Critical Care Medicine*	Retrospective cohort	TR-DGU (Trauma Registry of German Society for Trauma Surgery)	01–1993	12–2009	26.180	814	Both
21	Huber-Wagner et al. [20]	Germany, Austria and Switzerland	*Resuscitation*	Retrospective cohort	TRGTS (Trauma Registry of the German Trauma Society)	01–1993	12–2004	10.359	757	Both
22	Irfan et al. [34]	Qatar	*International Journal of Cardiology*	Retrospective cohort	Hamad Trauma Centre	01–2010	12–2015	718	410	Both
23	Israr et al. [42]	USA	*Injury*	Retrospective cohort	2 Lvl 1 hospitals in Arizona	02–2013	12–2017	277	277	Both
24	Javaudin et al. [46]	France	*Prehospital Emergency Care*	Retrospective cohort	French National OHCA Registry (RéAC)	07–2011	06–2018	82.125	2.981	Both
25	Jun et al. [47]	Korea	*Clinical and Experimental Emergency Medicine*	Retrospective cohort	Out-of-Hospital Cardiac Arrest Surveillance (OHCAS) of the KCDC	01–2012	12–2016	142.905	8.237	Both
26	Kitamura et al. [26]	Japan	*British Medical Journal Open*	Retrospective cohort	Utstein Osaka Project	01–2005	12–2011	47.735	2.065	Blunt
27	Lin et al. [25]	Taiwan	*Resuscitation*	Retrospective cohort	Utstein-style population database	01–2004	12–2010	3.607	424	Both
28	Lockey et al. [3]	UK	*Annals of Emergency Medicine*	Retrospective cohort	London HEMS database	07–1994	06–2004	12.086	909	Both
29	Lu et al. [43]	Taiwan	*Scandinavian Journal of Trauma, Resuscitation and Emergency Medicine*	Retrospective cohort	Local EMS database	01–2014	12–2016	4.526	560	Both
30	Moriwaki et al. [22]	Japan	*World Journal of Surgery*	Retrospective cohort	Local Hospital	2000	2010	477	477	Blunt
31	Pickens et al. [17]	USA	*Journal of Trauma: Injury, Infection, and Critical Care*	Retrospective cohort	Seattle Fire Department	01–1995	04–2001	266	184	Both
32	Stockinger et al. [16]	USA	*Journal of American College of Surgeons*	Retrospective cohort	Local hospital	01–1997	12–2002	25.489	588	Both
33	ter Avest et al. [44]	UK	*Resuscitation*	Retrospective cohort	KSSAAT (Kent, Surrey & Sussex Air Ambulance trust)	07–2013	05–2018	263	262	Both
34	Tsutsumi et al. [38]	Japan	*Injury*	Retrospective cohort	Japan Trauma Data Bank	01–2004	12–2015	236.698	4.313	Blunt
35	Willis et al. [18]	Australia	*Injury*	Retrospective cohort	VSTR (Victorian State Trauma System)	07–2001	12–2004	5.349	89	Both
36	Yamamoto et al. [45]	Japan	*Scandinavian Journal of Trauma, Resuscitation and Emergency Medicine*	Prospective observational study with post hoc analysis	Regional EMS database	01–2012	03–2013	16.452	1.030	Both

### Overall TCA mortality and neurological outcome

**Fig. 2:**
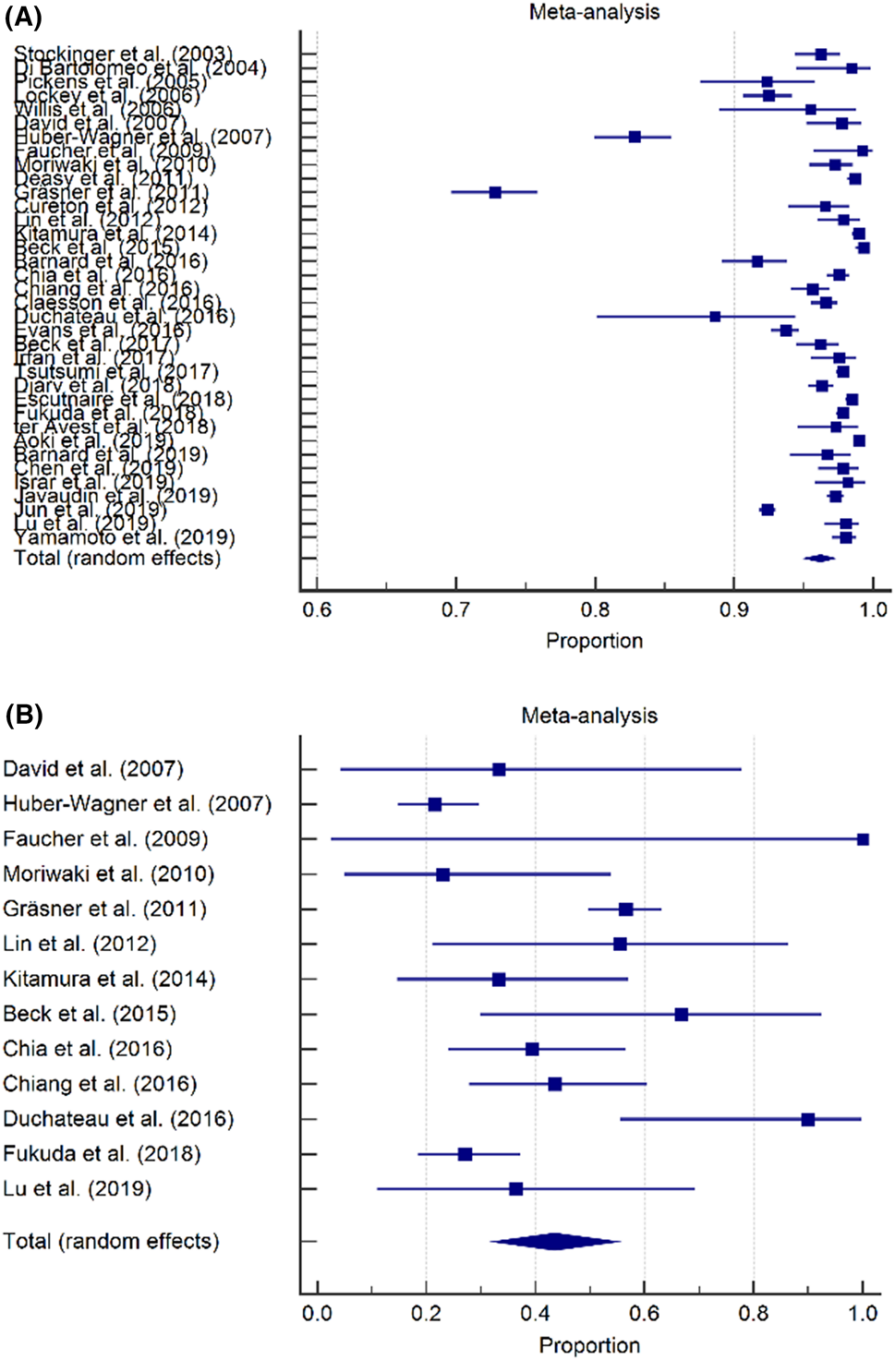
Overall TCA mortality and neurological outcome, forest plots. **A** Overall prehospital TCA mortality was 96.2% (95% CI 95.0–97.2). **B** Favorable neurological outcome was observed in 43.5% of the TCA survival patients (95% CI 32.3–55.0)

The overall pooled mortality rate for TCA was 96.2% (95% CI 95.0–97.2) (Fig. 2). Within the 36 included studies, 13 studies reported neurological outcome as defined in the methods section [1, 2, 19–22, 25, 26, 30, 31, 33, 37, 43]. Of all TCA survivors, a (pooled) favorable neurological outcome was observed in 43.5% of the TCA survival patients (95% CI 32.3–55.0).

### Impact of database registry type on TCA mortality

**Fig. 3:**
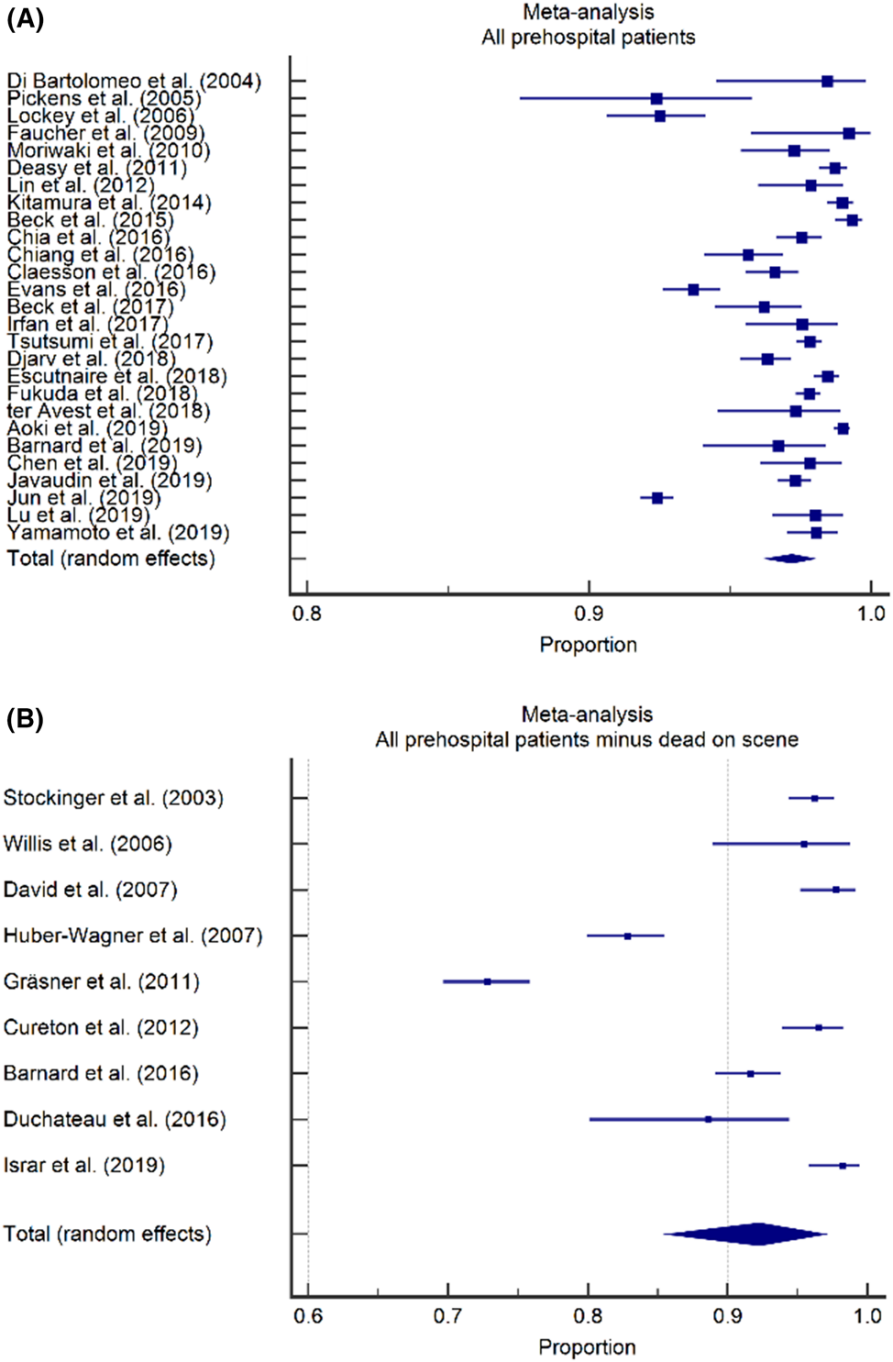
Impact of database registry type on TCA mortality, forest plots. **A** Overall mortality in studies including prehospital deaths was 97.2% (95% CI 96.3–98.0). **B** Overall mortality in studies excluding prehospital deaths was 92.3% (95% CI 85.7–96.3)

To investigate TCA mortality for different database registry types, pooled mortality rates were calculated separately for 27 studies including prehospital deaths [1, 3, 6, 17, 21, 22, 24–27, 29–32, 34–41, 43–47] and for nine studies excluding prehospital deaths (Fig. 3) [2, 16, 18–20, 23, 28, 33, 42]. The pooled mortality rates were 97.2% (95% CI 96.3–98.0) and 92.3% (95% CI 85.7–96.9) for studies including patients declared dead on-scene and for studies excluding patients declared dead on-scene, respectively.

### Impact of database registry type on neurological outcome

**Fig. 4:**
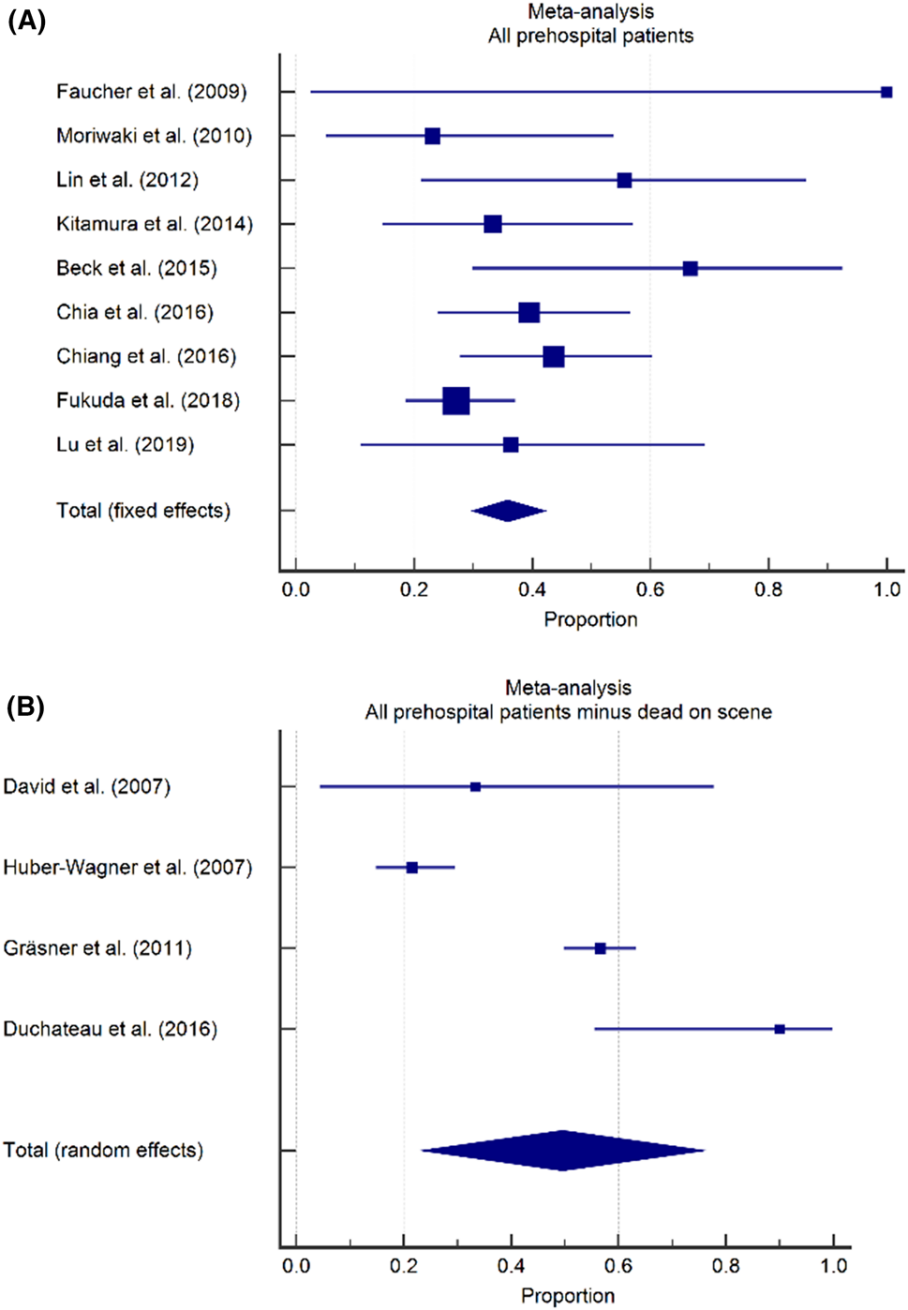
Impact of database registry type on neurological outcome, forest plots. **A** A favorable neurologic outcome was observed in 35.8% of survivors in studies including prehospital deaths (95% CI 29.8–42.2). **B** A favorable neurologic outcome was observed in 49.5% of survivors in studies excluding prehospital deaths (95% CI 23.3–75.9)

Likewise, neurological outcome was investigated for different database types by calculating the pooled proportion of surviving patients with a favorable neurological outcome for nine studies [21, 22, 25, 26, 29–31, 37, 43] including and for four studies excluding prehospital deaths separately (Fig. 4) [2, 19, 20, 33]. A favorable neurological outcome was seen in 35.8% (95% CI 29.8–42.2) of surviving patients in studies including prehospital deaths and in 49.5% (95% CI 23.3–75.9) of surviving patients in studies excluding prehospital deaths.

### Impact of organization of EMS system on TCA mortality

**Fig. 5:**
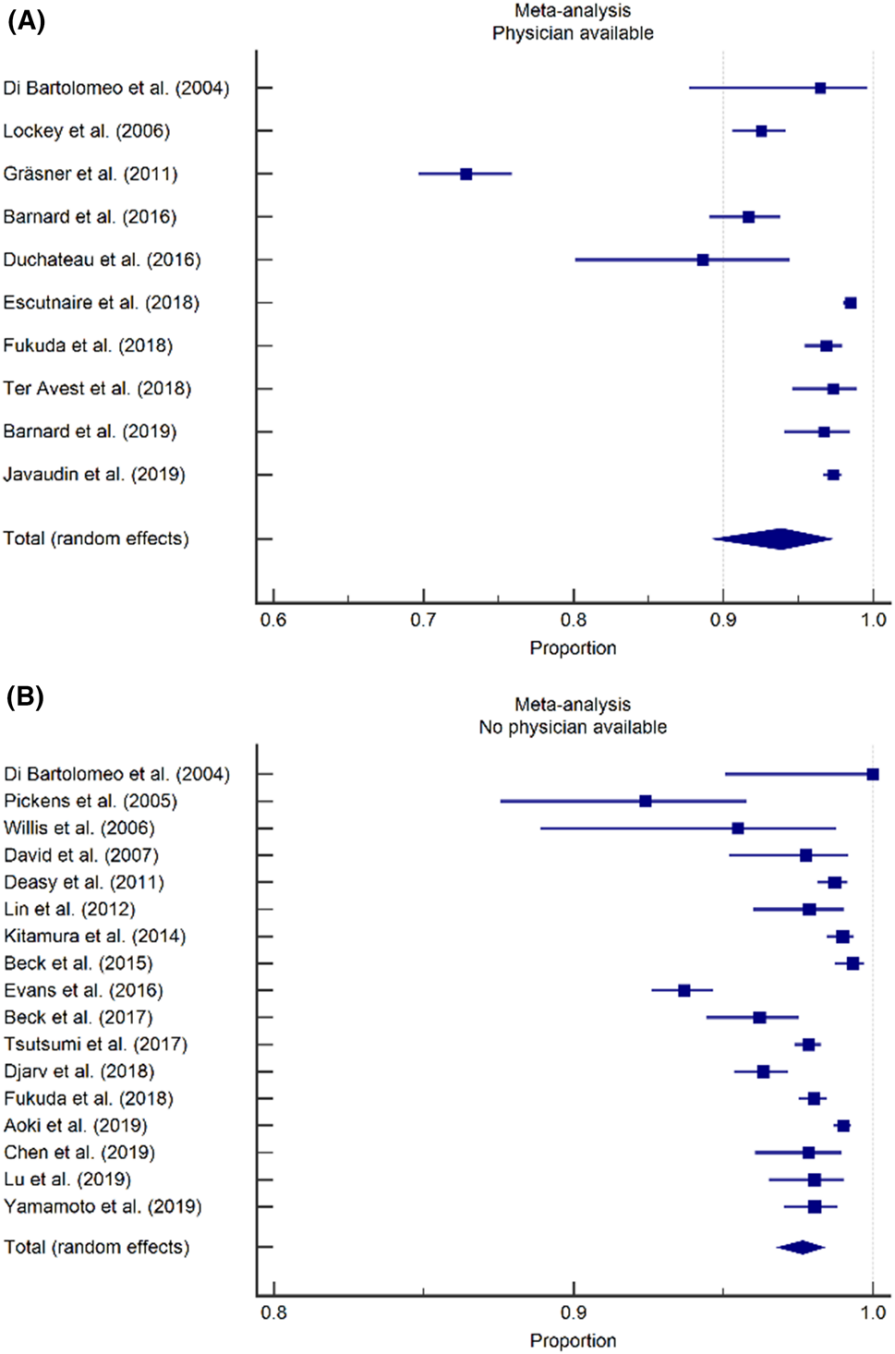
Impact of organization of EMS system on TCA mortality, forest plots. **A** Overall mortality in studies from countries or regions with a physician available on-scene was 93.9% (95% CI 89.3–97.2). **(B)** Overall mortality in studies from countries or regions without a physician available on-scene was 97.6% (95% CI 96.8–98.4)

To investigate mortality differences between EMS systems with and without a physician on scene, mortality rates were calculated separately for studies from countries or regions with and countries or regions without a physician-based service (Fig. 5). The pooled mortality rates were 93.9% (95% CI 89.3–97.2) in 10 studies where a physician was available at the prehospital scene [2, 3, 6, 28, 33, 36, 37, 40, 44, 46] and 97.6% (95% CI 96.8–98.4) in 17 studies where no physician was available at the prehospital scene [1, 6, 17–19, 24–27, 29, 35, 37–39, 41, 43, 45].

### Impact of organization of EMS system on neurological outcome

**Fig. 6:**
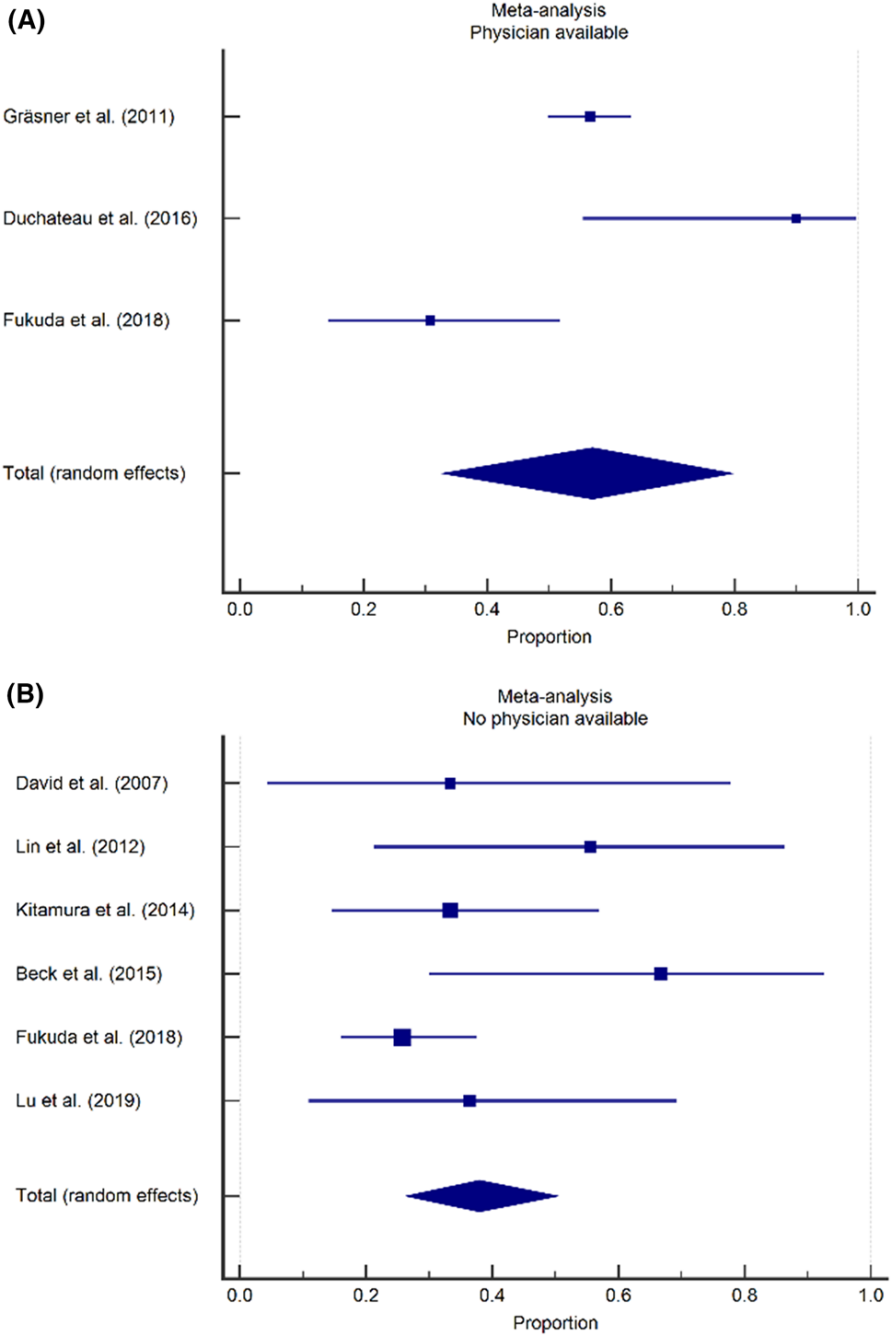
Impact of organization of EMS system on neurological outcome, forest plots. **A** A favorable neurologic outcome was observed in 57.0% of survivors in studies from regions with a physician available on scene (95% CI 32.8–79.6). **B** A favorable neurologic outcome was observed in 38.0% of survivors in studies from regions without a physician available on scene (95% CI 26.4–50.3)

Similarly, the proportion of surviving patients with a favorable neurologic status was calculated for studies from countries or regions with and countries or regions without a physician-based service. For three studies from a country or region with a physician-based EMS system [2, 33, 37], the pooled proportion of patients with a favorable neurologic outcome was 57.0% (95% CI 32.8–79.6). For six studies from a country or region without a physician-based EMS system, the pooled proportion of patients with a favorable neurologic outcome was 38.0% (95% CI 26.4–50.3) (Fig. 6) [1, 19, 25, 26, 37, 43].

### Prognostic factors for TCA mortality

**Fig. 7:**
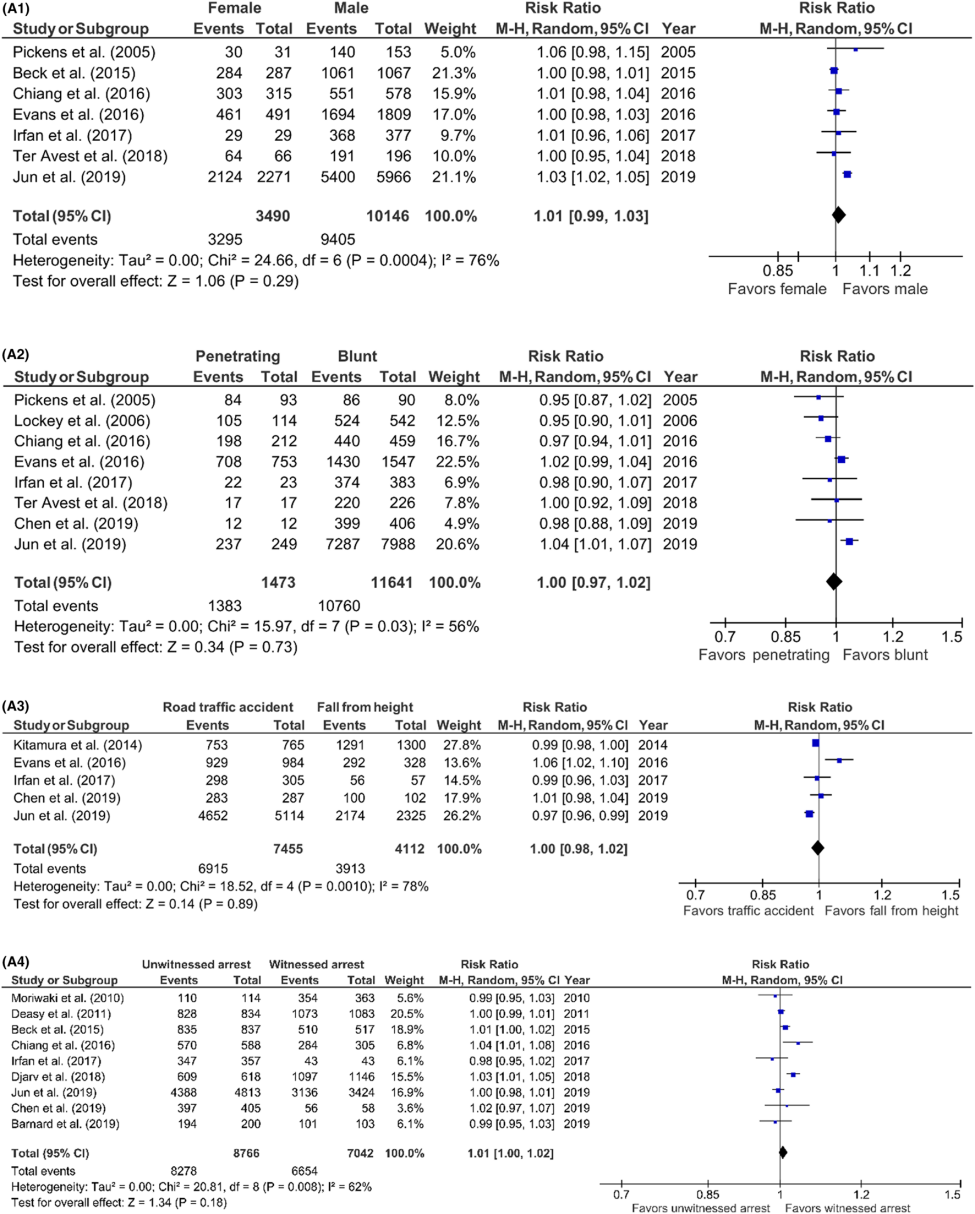

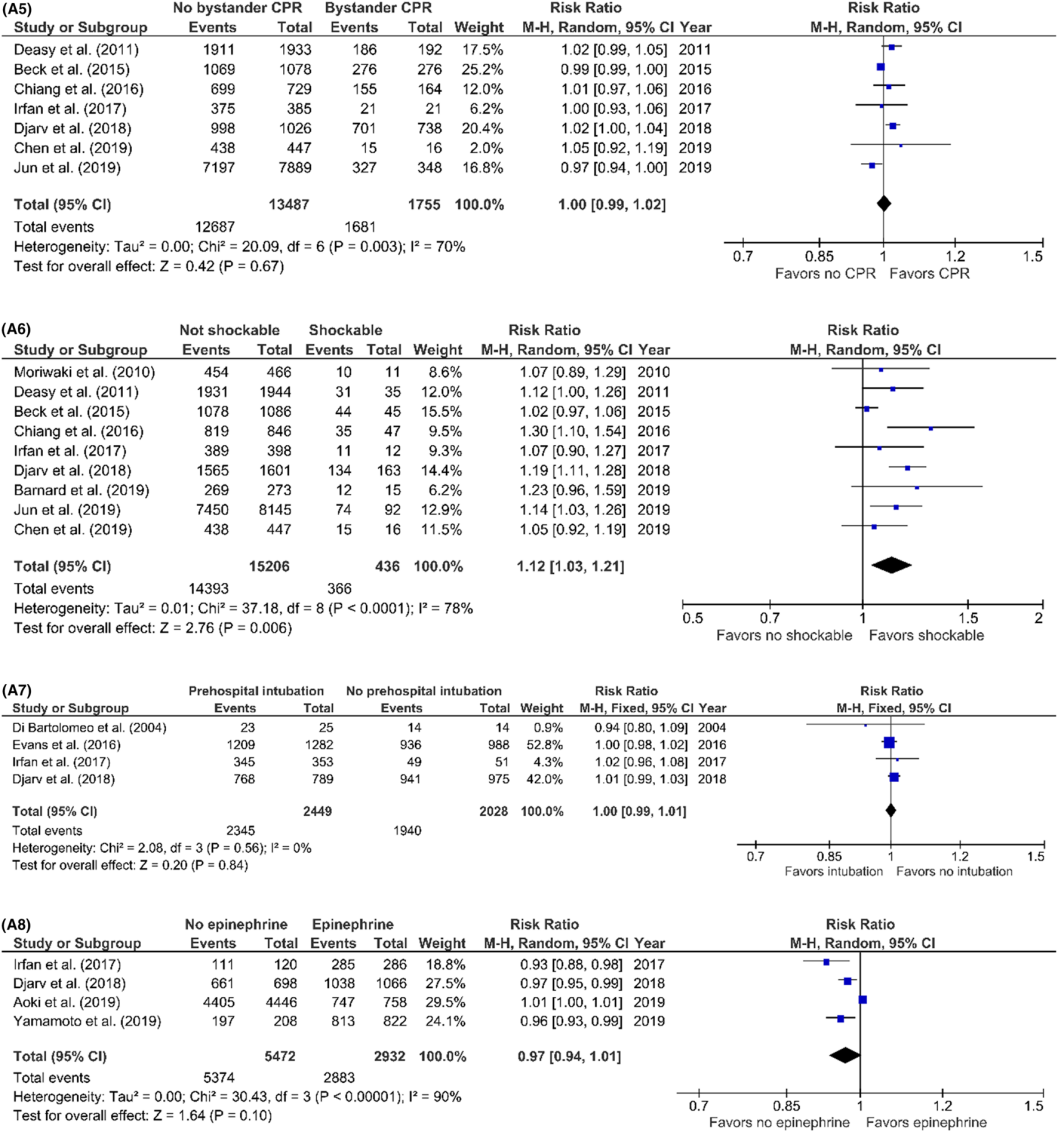

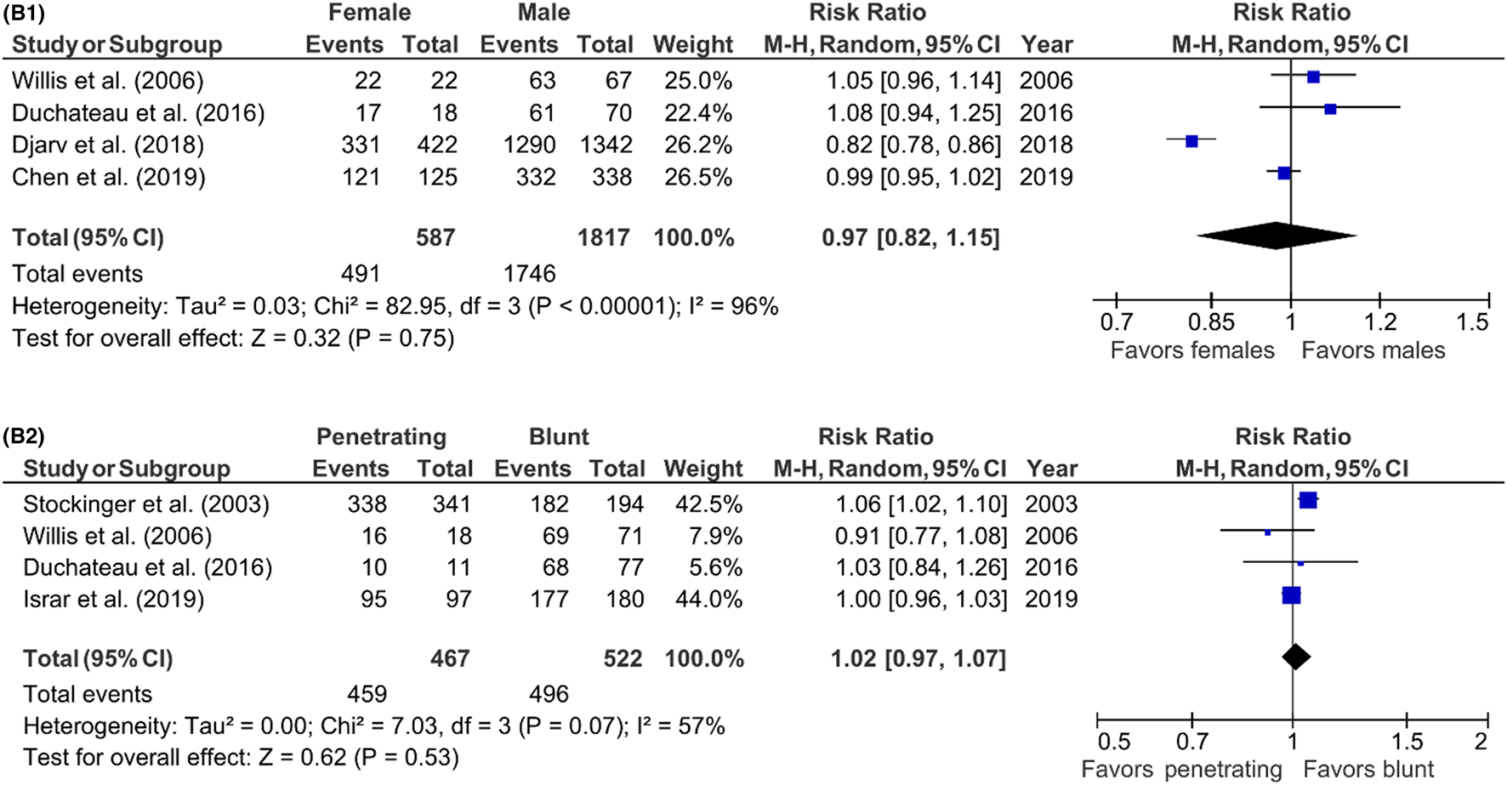
**A1–7A8** Predictors of mortality after prehospital TCA in studies including prehospital deaths, forest plots (*MH* Mantel–Haenszel, *CI* confidence interval). **A1** Sex (female vs. male). **A2** Trauma type (penetrating vs. blunt). **A3** Blunt trauma type (road traffic accident vs. fall from height). **A4** Witnessed arrest (unwitnessed vs. witnessed). **A5** Bystander CPR (no bystander CPR vs. bystander CPR). **A6** First monitored rhythm (not shockable vs. shockable). **A7** Prehospital intubation (prehospital intubation vs. no intubation). **A8** Prehospital administration of epinephrine (no epinephrine vs. epinephrine). **B1–7B2:** predictors of mortality after prehospital TCA in studies excluding prehospital deaths, forest plots (*MH* Mantel–Haenszel, *CI* confidence interval). **B1** Sex (female vs. male). **B2** Trauma type (penetrating vs. blunt)

Risk ratios of possible prognostic factors were calculated to investigate the association between pre- and intra-arrest factors and TCA mortality. Risk ratios were calculated for studies including prehospital deaths and for studies excluding prehospital deaths separately (Fig. 7) (Table 3; supplement). In studies including prehospital deaths, only the first monitored ECG-rhythm was associated with mortality (RR 1.12; 95% CI 1.03–1.21; *p* = 0.006). No risk factors were identified in studies excluding prehospital deaths. The evaluation of the funnel plots using Egger’s regression test showed that only in the analysis of the prognostic factor ‘sex’ in the studies excluding prehospital deaths, there might have been a significant amount of publication bias. The power analysis showed that all risk ratio analysis had sufficient power to support our conclusions; all prognostic factor analysis had a power of 1.00.

## Discussion

Circulatory arrest after trauma is a severe and life-threatening situation that mandates urgent action. Over the past years increased interest in this topic has led to broad recognition of this condition, with aggressive prehospital and emergency department resuscitation algorithms aimed at early treatment of reversible causes being introduced in prehospital and emergency department guidelines. This systematic review and meta-analysis provides a comprehensive overview of reported mortality rates after prehospital resuscitation of patients with cardiac arrest after trauma. In the current review on TCA in adult patients, the pooled mortality rate for traumatic cardiac arrest was 96.2% and a favorable neurological outcome was reported in 43.5% of surviving patients. A shockable first monitored ECG rhythm was the only patient related factor associated with a decreased risk of dying.

The results of the current systematic review and meta-analysis are in line with those of two other reviews that have been published on this subject in the last decade. Zwingmann et al. included 46 studies in a 2012 systematic review and reported a mortality rate of 96.7% among (mostly) adult patients with TCA [4]. A favorable neurologic outcome was reported in 44.3% of survivors. A more recent meta-analysis of factors associated with survival after TCA by Tran et al. did not report a pooled mortality rate, but did find first ECG rhythm and the presence of cardiac motion on ultrasound to be associated with a decreased risk to die [5].

The current study adds two important findings to the existing body of literature. At first, pooled mortality rates varied among studies based on the inclusion or exclusion of patients that had deceased on-scene. Studies excluding prehospital deaths show an almost three-fold higher proportion of patients surviving compared to studies including prehospital deaths. While this is hardly surprising, we believe this is an important factor to consider when interpreting studies on prehospital TCA that is often overlooked in discussions on TCA. Indeed, in a recent study from our own country (published after the search for this review), survival was 3.9% when including patients that had deceased on-scene and 10.9% when these patients were excluded [48].

Second, studies from EMS systems where a physician had been available at the prehospital scene had a trend towards a lower pooled mortality rate (93.9%; 95% CI 89.3–97.2) than studies from EMS systems where no physician was available on-scene (96.8%; 95% CI 96.8–98.4%), with an almost two-fold increase in survival in the former category. While the available data did not allow for a meta-analysis of this particular factor, individual studies suggest that the presence of a physician on-scene (and thus the availability of advanced life support interventions such as drug assisted intubation, finger- or tube thoracostomy or thoracotomy, transfusion of blood products, vasopressor drugs) is associated with increased odds of survival in TCA patients [6, 40]. While a part of this effect should be attributed to effective field-triage (with physicians only arriving on-scene when ROSC has already been obtained and physicians dispatch being cancelled in the most severe cases), we do strongly believe that certain patients do benefit from these aggressive resuscitative measures on-scene.

The big question that has not been answered yet, is how to identify those patients who do benefit from on-scene advanced life support, and conversely, those patients who should be transported to a nearby trauma center without any delay. With mortality rates consistently ranging above 90% and a significant proportion of patients having an unfavorable neurologic outcome, everyone will agree that neurological intact survival after TCA is still exceptional. It would therefore be helpful if resource intensive prehospital (and in-hospital) resuscitation attempts could be preserved for those with realistic odds of survival. The most recent ERC guidelines do provide some guidance and we believe these should be adapted to fit individual EMS systems; if reversible causes for TCA can be promptly and effectively treated on-scene, these should be looked for and treated accordingly as an integral part of resuscitation. Examples include the immediate treatment of cardiac tamponade by resuscitative thoracotomy or needle/finger thoracostomy in tension-pneumothorax [49–51]. If the level of training of the emergency care provider does not allow for such procedures or if the injuries leading to cardiac arrest cannot be adequately addressed on-scene (for instance hypovolemia due to penetrating truncal injury), no time should be wasted on any on-scene interventions and the patient should be transported to the nearest trauma center without delay.

This study has several limitations. Next to the factors investigated in this study, factors such as on-scene time, time to ROSC, and distance to a trauma center are probably important determinants of survival as well. However, these data are seldom available in any registry or database and almost never published. Such and other confounding factors make it difficult to calculate and interpret the prognostic value of intra-arrest factors or the value of resuscitative measures. In addition, the way mortality and survival rates, neurological outcome rates, prognostic factors, et cetera, are reported, differs strongly among studies. We advocate future studies to comply with the Utstein consensus statement on reporting outcomes after out of hospital cardiac arrest [52]. Finally, some primary studies used in our systematic review and meta-analysis used the same or a similar database as other primary studies, with an overlapping inclusion period as well. As a result, it is possible that some patients have been included in our systematic review and meta-analysis several times.

### Conclusion

In conclusion, prehospital TCA is associated with a high mortality rate, with approximately one in twenty patients surviving to discharge. When interpreting results from studies on this subject, factors such as the in- or exclusion of patients that have deceased on-scene and the type of prehospital EMS system (physician-based) should be considered. Apart from first monitored ECG rhythm, this study found no other prognostic factors available to differentiate between survivors and non-survivors.

### Electronic supplementary material

Below is the link to the electronic supplementary material.

Supplementary file1 Table 3 (supplement): Predictors of mortality after prehospital TCA (CI = Confidence Interval). (DOCX 17 kb)

Supplementary file2 Figure 8 (supplement): Overall TCA mortality and neurological outcome, funnel plots. (A): Overall prehospital TCA mortality was 96.2% (95% CI 95.0-97.2). (B) Favorable neurological outcome was observed in 43.5% of the TCA survival patients (95% CI 32.3-55.0). (TIF 50 kb)

Supplementary file3 (TIF 49 kb)

Supplementary file4 Figure 9 (supplement): Impact of database registry type on TCA mortality, funnel plots. (A) Overall mortality in studies including prehospital deaths was 97.2% (95% CI 96.3-98.0). (B) Overall mortality in studies excluding prehospital deaths was 92.3% (95% CI 85.7-96.3). (TIF 60 kb)

Supplementary file5 (TIF 49 kb)

Supplementary file6 Figure 10 (Supplement): Impact of database registry type on neurological outcome, funnel plots. (A): A favorable neurologic outcome was observed in 35.8% of survivors in studies including prehospital deaths (95% CI 29.8-42.2). (B) A favorable neurologic outcome was observed in 49.5% of survivors in studies excluding prehospital deaths (95% CI 23.3-75.9). (TIF 59 kb)

Supplementary file7 (TIF 47 kb)

Supplementary file8 Figure 11 (supplement): Impact of organization of EMS system on TCA mortality, funnel plots. (A) Overall mortality in studies from countries or regions with a physician available on-scene was 93.9% (95% CI 89.3-97.2). (B) Overall mortality in studies from countries or regions without a physician available on-scene was 97.6% (95% CI 96.8-98.4). (TIF 56 kb)

Supplementary file9 (TIF 55 kb)

Supplementary file10 Figure 12 (supplement): Impact of organization of EMS system on neurological outcome, funnel plots. (A): A favorable neurologic outcome was observed in 57.0% of survivors in studies from regions with a physician available on scene (95% CI 32.8-79.6). (B) A favorable neurologic outcome was observed in 38.0% of survivors in studies from regions without a physician available on scene (95% CI 26.4-50.3). (TIF 57 kb)

Supplementary file11 (TIF 53 kb)

Supplementary file12 Figure 13A1-13A8 (supplement): Predictors of mortality after prehospital TCA in studies including prehospital deaths, funnel plots (A1) Sex (female vs. male). (A2) Trauma type (penetrating vs. blunt). (A3) Blunt trauma type (road traffic accident vs. fall from height). (A4) Witnessed arrest (unwitnessed vs. witnessed). (A5) Bystander CPR (no bystander CPR vs. bystander CPR). (A6) First monitored rhythm (not shockable vs. shockable). (A7) Prehospital intubation (prehospital intubation vs. no intubation). (A8) Prehospital administration of epinephrine (no epinephrine vs. epinephrine). (TIF 7 kb)

Supplementary file13 (TIF 7 kb)

Supplementary file14 (TIF 7 kb)

Supplementary file15 (TIF 7 kb)

Supplementary file16 (TIF 7 kb)

Supplementary file17 (TIF 7 kb)

Supplementary file18 (TIF 7 kb)

Supplementary file19 (TIF 7 kb)

Supplementary file20 Figure 13B1-13B2 (supplement): Predictors of mortality after prehospital TCA in studies excluding prehospital deaths, funnel plots. (B1) Sex (female vs. male). (B2) Trauma type (penetrating vs. blunt). (PNG 3 kb)

Supplementary file21 (PNG 3 kb)

Supplementary file22 Figure 14 (supplement): RevMan Risk of Bias Summary Tool results. A green plus indicated a low chance on bias. An empty box indicates an unknown chance on bias. (TIF 108 kb)

## Appendix

### Search terms

#### Embase.com

('out of hospital cardiac arrest'/de OR 'traumatic cardiac arrest'/de OR 'traumatic out of hospital cardiac arrest'/de OR 'traumatic cardiopulmonary arrest'/de OR (('air medical transport'/de OR helicopter/de OR 'ambulance'/exp) AND ('heart arrest'/de OR 'cardiopulmonary arrest'/de)) OR (((out-of-hospital OR prehospital* OR preclinical* OR bystander* OR ambulance* OR helicopter* OR mobile* OR trauma*) NEAR/6 (cardiac OR cardiopulmon* OR heart) NEAR/3 (arrest* OR resuscitat*)) OR ((out-of-hospital OR prehospital* OR preclinical* OR bystander* OR ambulance* OR helicopter* OR mobile* OR trauma*) NEAR/3 (CPR OR advanced-life-support* OR als)) OR ohca):ab,ti) AND ('injury'/de OR 'abdominal injury'/exp OR 'accidental injury'/exp OR 'barotrauma'/exp OR 'blood vessel injury'/exp OR 'blunt trauma'/exp OR 'burn'/exp OR 'chemical injury'/exp OR 'crush trauma'/exp OR 'drowning'/exp OR 'electric injury'/exp OR 'head and neck injury'/exp OR 'limb injury'/exp OR 'multiple trauma'/exp OR 'musculoskeletal injury'/exp OR 'organ injury'/exp OR 'pelvis injury'/exp OR 'perforation'/exp OR 'respiratory tract injury'/exp OR 'rupture'/exp OR 'seatbelt injury'/exp OR 'strangulation'/exp OR 'thorax injury'/exp OR 'traumatic amputation'/exp OR 'traumatic hematoma'/exp OR 'traumatic shock'/exp OR 'wound'/exp OR 'accident'/exp OR 'accidental injury'/de OR 'traumatic cardiac arrest'/de OR 'traumatic cardiac arrest'/de OR 'traumatic out of hospital cardiac arrest'/de OR 'traumatic cardiopulmonary arrest'/de OR ((trauma* NOT (non-trauma*)) OR accident* OR drowning OR (injur* NOT (kidney- injur*)) OR penetrat* OR blunt*):ab,ti) NOT ([Conference Abstract]/lim) AND [English]/lim NOT ([animals]/lim NOT [humans]/lim) NOT (juvenile/exp NOT adult/exp).

#### Medline Ovid

(Out-of-Hospital Cardiac Arrest/ OR ((Air Ambulances/ OR Ambulances/) AND (Heart Arrest/)) OR (((out-of-hospital OR prehospital* OR preclinical* OR bystander* OR ambulance* OR helicopter* OR mobile* OR trauma*) ADJ6 (cardiac OR cardiopulmon* OR heart) ADJ3 (arrest* OR resuscitat*)) OR ((out-of-hospital OR prehospital* OR preclinical* OR bystander* OR ambulance* OR helicopter* OR mobile* OR trauma*) ADJ3 (CPR OR advanced-life-support* OR als)) OR ohca).ab,ti.) AND ("Wounds and Injuries"/ OR exp Abdominal Injuries/ OR exp Amputation, Traumatic/ OR exp Arm Injuries/ OR exp Asphyxia/ OR exp Barotrauma/ OR exp Burns/ OR exp Crush Injuries/ OR exp Drowning/ OR exp Electric Injuries/ OR exp Fractures, Bone/ OR exp Leg Injuries/ OR exp Multiple Trauma/ OR exp Neck Injuries/ OR exp Occupational Injuries/ OR exp Shock, Traumatic/ OR exp Spinal Cord Injuries/ OR exp Thoracic Injuries/ OR exp Trauma, Nervous System/ OR exp Vascular System Injuries/ OR exp Wounds, Nonpenetrating/ OR exp Wounds, Penetrating/ OR ((trauma* NOT (non-trauma*)) OR accident* OR drowning OR (injur* NOT (kidney- injur*)) OR penetrat* OR blunt*).ab,ti.) AND english.la. NOT (exp animals/ NOT humans/) NOT ((exp child/ OR exp infant/ OR adolescent/) NOT exp adult/).

#### Web of science

TS=(((((out-of-hospital OR prehospital* OR preclinical* OR bystander* OR ambulance* OR helicopter* OR mobile* OR trauma*) NEAR/5 (cardiac OR cardiopulmon* OR heart) NEAR/2 (arrest* OR resuscitat*)) OR ((out-of-hospital OR prehospital* OR preclinical* OR bystander* OR ambulance* OR helicopter* OR mobile* OR trauma*) NEAR/2 (CPR OR advanced-life-support* OR als)) OR ohca)) AND (((trauma* NOT (non-trauma*)) OR accident* OR drowning OR (injur* NOT (kidney- injur*)) OR penetrat* OR blunt*)) NOT ((animal* OR rat OR rats OR mouse OR mice OR murine OR dog OR dogs OR canine OR cat OR cats OR feline OR rabbit OR cow OR cows OR bovine OR rodent* OR sheep OR ovine OR pig OR swine OR porcine OR veterinar* OR chick* OR zebrafish* OR baboon* OR nonhuman* OR primate* OR cattle* OR goose OR geese OR duck OR macaque* OR avian* OR bird* OR fish*) NOT (human* OR patient* OR women OR woman OR men OR man)) NOT ((juvenile* OR child* OR infant* OR adolescen*) NOT (adult*))) AND DT=(article) AND LA=(english).

#### Cochrane CENTRAL

((((out NEXT of NEXT hospital OR prehospital* OR preclinical* OR bystander* OR ambulance* OR helicopter* OR mobile* OR trauma*) NEAR/6 (cardiac OR cardiopulmon* OR heart) NEAR/3 (arrest* OR resuscitat*)) OR ((out NEXT of NEXT hospital OR prehospital* OR preclinical* OR bystander* OR ambulance* OR helicopter* OR mobile* OR trauma*) NEAR/3 (CPR OR advanced NEXT life NEXT support* OR als)) OR ohca):ab,ti) AND (((trauma* NOT (non NEXT trauma*)) OR accident* OR drowning OR (injur* NOT (kidney NEXT injur*)) OR penetrat* OR blunt*):ab,ti).

#### Google Scholar

"prehospital|preclinical|bystander|ambulance|traumatic cardiac|cardiopulmon|heart arrest|resuscitation" traumatic|accident|drowning|penetrating|blunt.
