# Correction to: Prehospital traumatic cardiac arrest: a systematic review and meta-analysis

**DOI:** 10.1007/s00068-022-01975-2

**Published:** 2022-05-05

**Authors:** Niek Johannes Vianen, Esther Maria Maartje Van Lieshout, Iscander Michael Maissan, Wichor Matthijs Bramer, Dennis Den Hartog, Michael Herman Jacob Verhofstad, Mark Gerrit Van Vledder

**Affiliations:** 1 Trauma Research Unit, Department of Surgery, Erasmus MC, University Medical Center Rotterdam, P.O. Box 2040, 3000 CA Rotterdam, The Netherlands; 2 Department of Anesthesiology, Erasmus University Medical Center Rotterdam, Rotterdam, The Netherlands; 3 Medical Library, Erasmus MC, Erasmus University Medical Centre Rotterdam, 3000 CS Rotterdam, The Netherlands

## Correction to: European Journal of Trauma and Emergency Surgery 10.1007/s00068-022-01941-y

The original version of this article unfortunately contained mistakes.

In this article the author name “Iscander Michael Maissan” was incorrectly written as “Iscander Maria Maissan”.

In the abstract Section "Results", sixth sentence should read:

Favorable neurological outcome rates were 57.0% if a physician was available on scene and 38.0% if no physician was available.

The original article has been corrected.

