# Supplementary material for: Prehospital traumatic cardiac arrest: a systematic review and meta-analysis
Source: Eur J Trauma Emerg Surg. 2022 Mar 25;48(4):3357–72. doi: 10.1007/s00068-022-01941-y (PMC9360068; doi:10.1007/s00068-022-01941-y)
Supplement: Supplementary file 1 — Supplementary file1 Table 3 (supplement): Predictors of mortality after prehospital TCA (CI = Confidence Interval). (DOCX 17 kb) [file 68_2022_1941_MOESM1_ESM.docx]

| **Table 3** | | | | |
| --- | --- | --- | --- | --- |
| **Studies including prehospital deaths** | | | | |
|  |  |  | **Including prehospital deaths** | |
| **Prognostic factor** | **Intervention group** | **Control group** | **Risk ratio and 95% CI** | **P-value** |
| A1) Sex | Female | Male | 1.01 (0.99 – 1.03) | 0.67 |
| A2) Trauma type | Penetrating | Blunt | 1.00 (0.97 - 1.02) | 0.86 |
| A3) Blunt trauma type | Road traffic accident | Fall from height | 1.00 (0.98 - 1.02) | 0.68 |
| A4) Witnessed arrest | Unwitnessed arrest | Witnessed arrest | 1.01 (1.00 - 1.02) | 0.18 |
| A5) Bystander CPR | No bystander CPR | Bystander CPR | 1.00 (0.99 - 1.02) | 0.67 |
| A6) First monitored rhythm | Not shockable | Shockable | 1.12 (1.03 - 1.21) | 0.006 |
| A7) Prehospital intubation | Prehospital intubation | No prehospital intubation | 1.00 (0.99 - 1.01) | 0.84 |
| A8) Prehospital administration of epinephrine | No epinephrine | Epinephrine | 0.97 (0.94 - 1.01) | 0.10 |
|  | | | | |
| **Studies excluding prehospital deaths** | | | | |
| **Prognostic factor** | **Intervention group** | **Control group** | **Risk ratio** | **P-value** |
| B1) Sex | Female | Male | 0.97 (0.82 - 1.15) | 0.75 |
| B2) Trauma type | Penetrating | Blunt | 1.02 (0.97 - 1.07) | 0.53 |
| B3) Blunt trauma type | Insufficient data for analysis | | | |
| B4) Witnessed arrest | Insufficient data for analysis | | | |
| B5) Bystander CPR | Insufficient data for analysis | | | |
| B6) First monitored rhythm | Insufficient data for analysis | | | |
| B7) Prehospital intubation | Insufficient data for analysis | | | |
| B8) Prehospital administration of epinephrine | Insufficient data for analysis | | | |
