# Supplementary figures and images for: Prehospital traumatic cardiac arrest: a systematic review and meta-analysis
Source: Eur J Trauma Emerg Surg. 2022 Mar 25;48(4):3357–72. doi: 10.1007/s00068-022-01941-y (PMC9360068; doi:10.1007/s00068-022-01941-y)

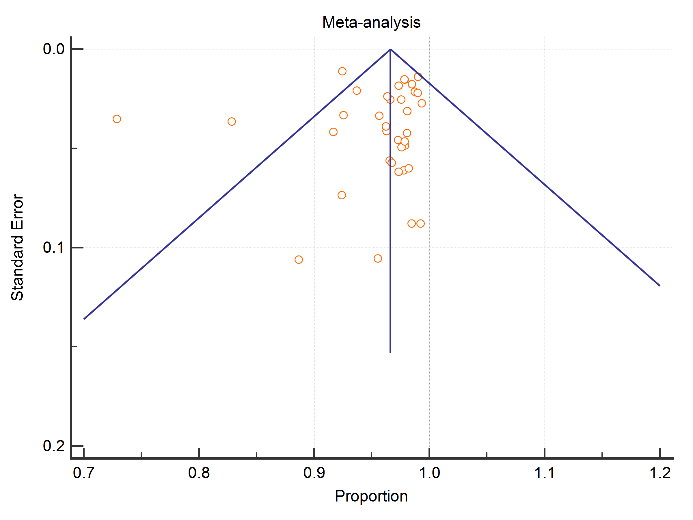

Supplement: Supplementary file 2 — Supplementary file2 Figure 8 (supplement): Overall TCA mortality and neurological outcome, funnel plots. (A): Overall prehospital TCA mortality was 96.2% (95% CI 95.0-97.2). (B) Favorable neurological outcome was observed in 43.5% of the TCA survival patients (95% CI 32.3-55.0). (TIF 50 kb) [file 68_2022_1941_MOESM2_ESM.tif]

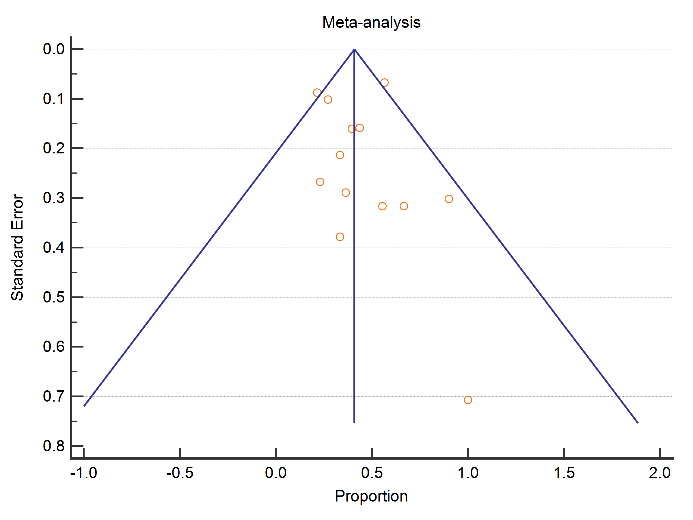

Supplement: Supplementary file 3 — Supplementary file3 (TIF 49 kb) [file 68_2022_1941_MOESM3_ESM.tif]

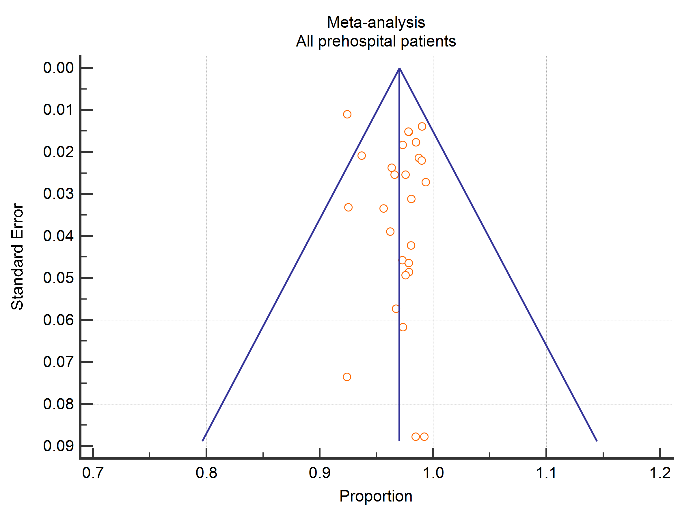

Supplement: Supplementary file 4 — Supplementary file4 Figure 9 (supplement): Impact of database registry type on TCA mortality, funnel plots. (A) Overall mortality in studies including prehospital deaths was 97.2% (95% CI 96.3-98.0). (B) Overall mortality in studies excluding prehospital deaths was 92.3% (95% CI 85.7-96.3). (TIF 60 kb) [file 68_2022_1941_MOESM4_ESM.tif]

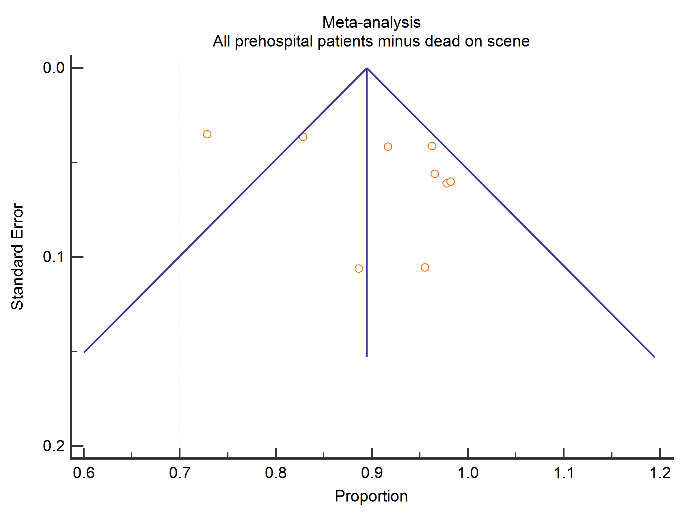

Supplement: Supplementary file 5 — Supplementary file5 (TIF 49 kb) [file 68_2022_1941_MOESM5_ESM.tif]

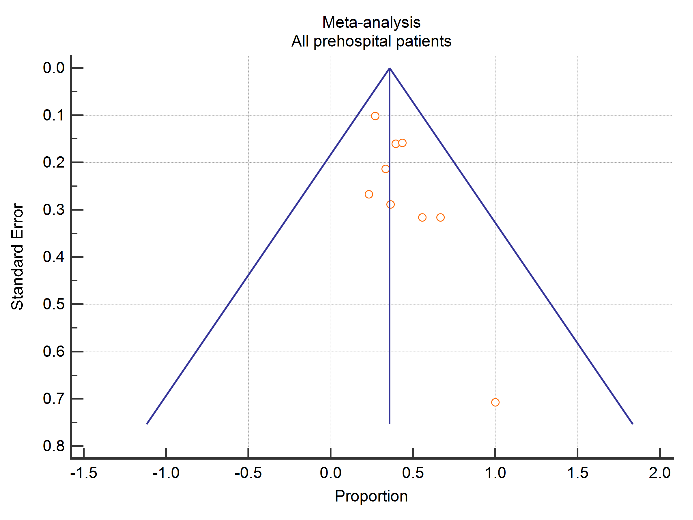

Supplement: Supplementary file 6 — Supplementary file6 Figure 10 (Supplement): Impact of database registry type on neurological outcome, funnel plots. (A): A favorable neurologic outcome was observed in 35.8% of survivors in studies including prehospital deaths (95% CI 29.8-42.2). (B) A favorable neurologic outcome was observed in 49.5% of survivors in studies excluding prehospital deaths (95% CI 23.3-75.9). (TIF 59 kb) [file 68_2022_1941_MOESM6_ESM.tif]

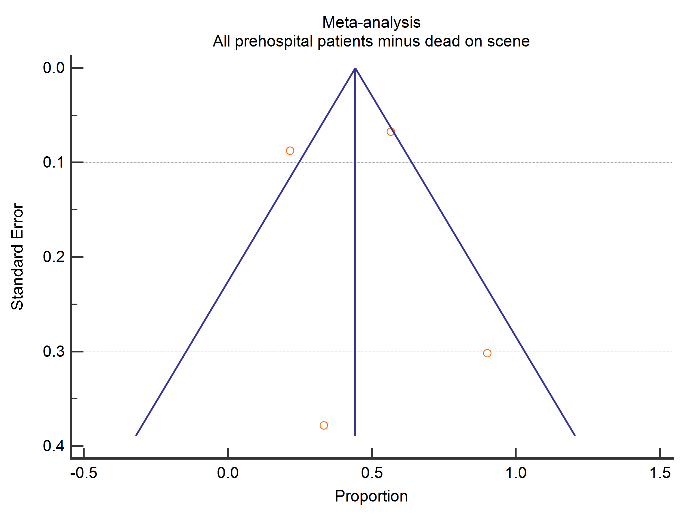

Supplement: Supplementary file 7 — Supplementary file7 (TIF 47 kb) [file 68_2022_1941_MOESM7_ESM.tif]

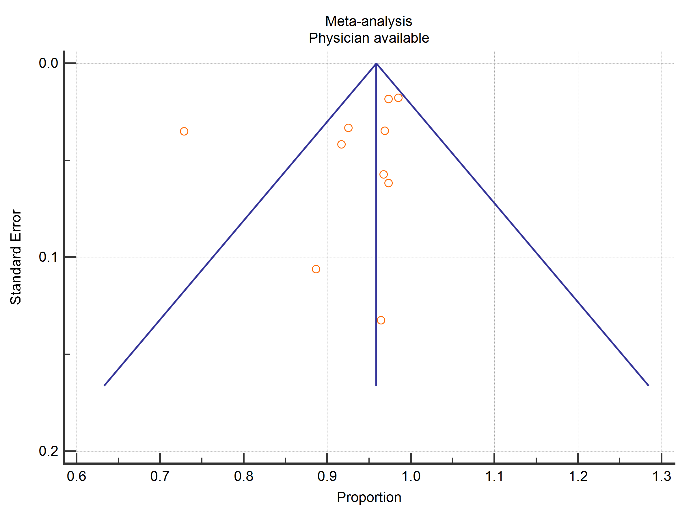

Supplement: Supplementary file 8 — Supplementary file8 Figure 11 (supplement): Impact of organization of EMS system on TCA mortality, funnel plots. (A) Overall mortality in studies from countries or regions with a physician available on-scene was 93.9% (95% CI 89.3-97.2). (B) Overall mortality in studies from countries or regions without a physician available on-scene was 97.6% (95% CI 96.8-98.4). (TIF 56 kb) [file 68_2022_1941_MOESM8_ESM.tif]

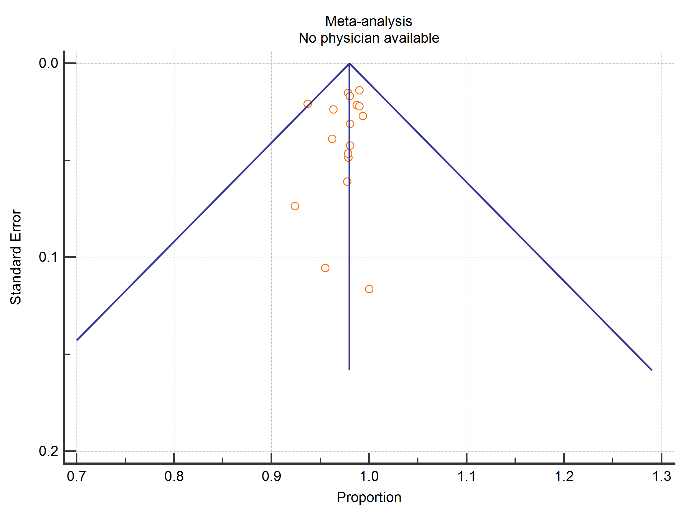

Supplement: Supplementary file 9 — Supplementary file9 (TIF 55 kb) [file 68_2022_1941_MOESM9_ESM.tif]

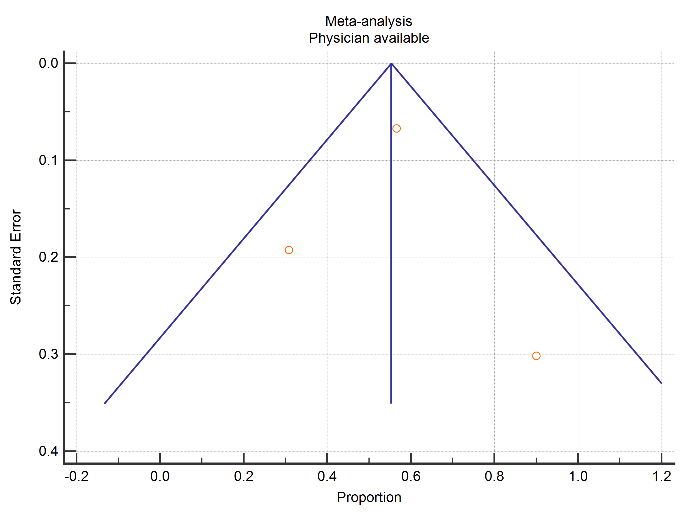

Supplement: Supplementary file 10 — Supplementary file10 Figure 12 (supplement): Impact of organization of EMS system on neurological outcome, funnel plots. (A): A favorable neurologic outcome was observed in 57.0% of survivors in studies from regions with a physician available on scene (95% CI 32.8-79.6). (B) A favorable neurologic outcome was observed in 38.0% of survivors in studies from regions without a physician available on scene (95% CI 26.4-50.3). (TIF 57 kb) [file 68_2022_1941_MOESM10_ESM.tif]

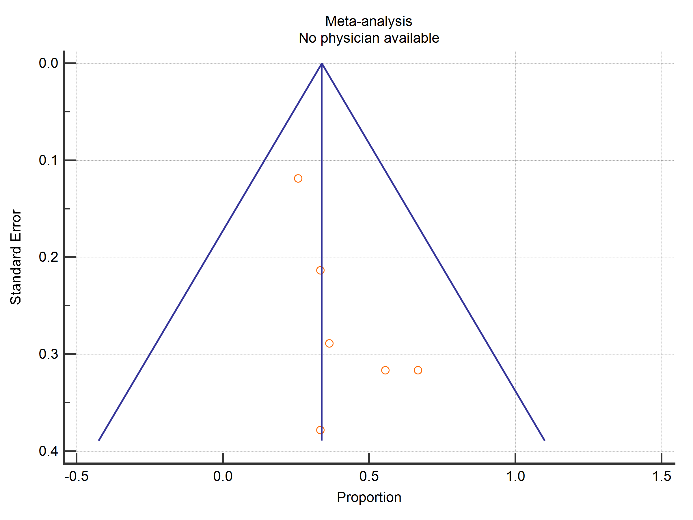

Supplement: Supplementary file 11 — Supplementary file11 (TIF 53 kb) [file 68_2022_1941_MOESM11_ESM.tif]

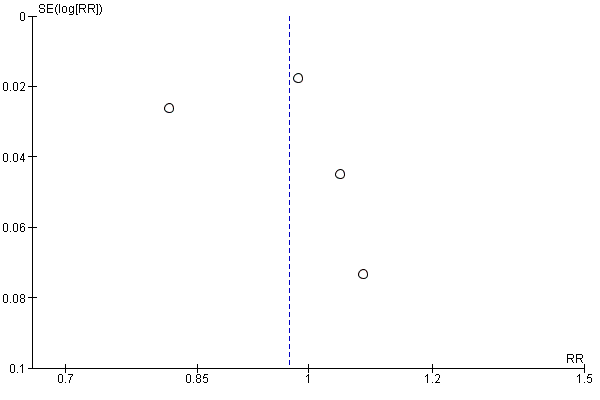

Supplement: Supplementary file 20 — Supplementary file20 Figure 13B1-13B2 (supplement): Predictors of mortality after prehospital TCA in studies excluding prehospital deaths, funnel plots. (B1) Sex (female vs. male). (B2) Trauma type (penetrating vs. blunt). (PNG 3 kb) [file 68_2022_1941_MOESM20_ESM.png]

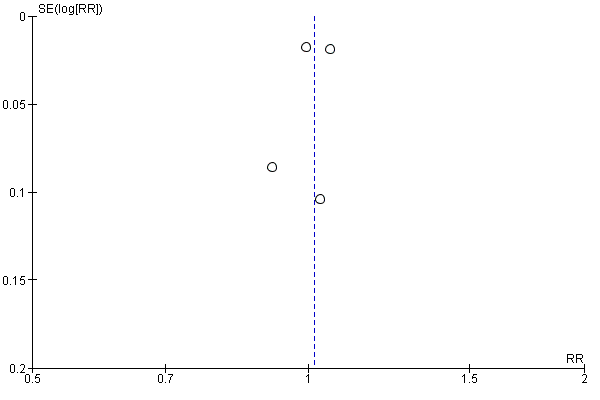

Supplement: Supplementary file 21 — Supplementary file21 (PNG 3 kb) [file 68_2022_1941_MOESM21_ESM.png]

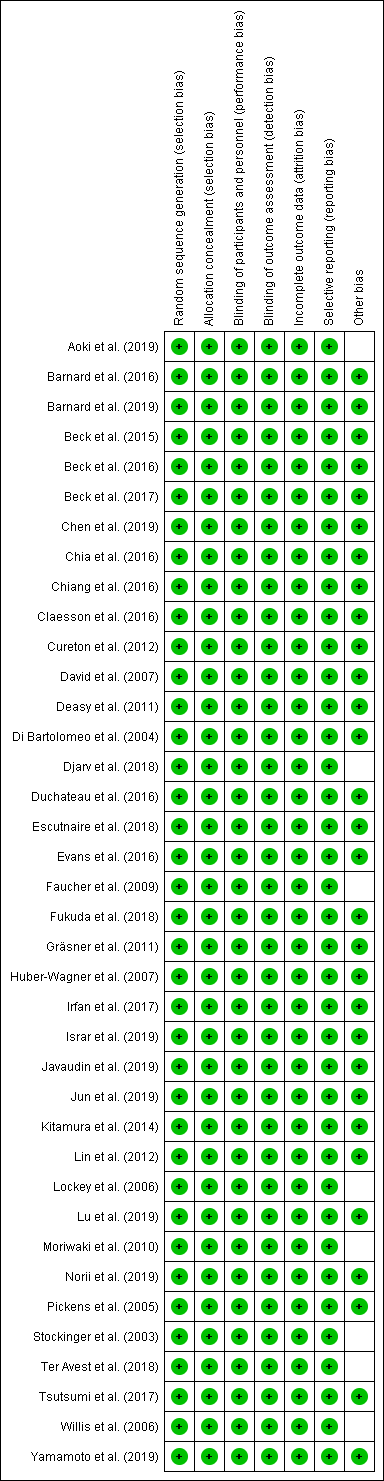

Supplement: Supplementary file 22 — Supplementary file22 Figure 14 (supplement): RevMan Risk of Bias Summary Tool results. A green plus indicated a low chance on bias. An empty box indicates an unknown chance on bias. (TIF 108 kb) [file 68_2022_1941_MOESM22_ESM.tif]
